# Obesity and Surgical Wound Healing: A Current Review

**DOI:** 10.1155/2014/638936

**Published:** 2014-02-20

**Authors:** Yvonne N. Pierpont, Trish Phuong Dinh, R. Emerick Salas, Erika L. Johnson, Terry G. Wright, Martin C. Robson, Wyatt G. Payne

**Affiliations:** ^1^Institute for Tissue Regeneration, Repair & Rehabilitation, Bay Pines VA Health Care System, Bay Pines, FL 33744, USA; ^2^Division of Plastic Surgery, University of South Florida, Tampa, FL 33620, USA

## Abstract

*Objective*. The correlation between obesity and deficient wound healing has long been established. This review examines the current literature on the mechanisms involved in obesity-related perioperative morbidity. *Methods*. A literature search was performed using Medline, PubMed, Cochrane Library, and Internet searches. Keywords used include obesity, wound healing, adipose healing, and bariatric and surgical complications. *Results*. Substantial evidence exists demonstrating that obesity is associated with a number of postoperative complications. Specifically in relation to wound healing, explanations include inherent anatomic features of adipose tissue, vascular insufficiencies, cellular and composition modifications, oxidative stress, alterations in immune mediators, and nutritional deficiencies. Most recently, advances made in the field of gene array have allowed researchers to determine a few plausible alterations and deficiencies in obese individuals that contribute to their increased risk of morbidity and mortality, especially wound complications. *Conclusion*. While the literature discusses how obesity may negatively affect health on various of medical fronts, there is yet to be a comprehensive study detailing all the mechanisms involved in obesity-related morbidities in their entirety. Improved knowledge and understanding of obesity-induced physiological, cellular, molecular, and chemical changes will facilitate better assessments of surgical risks and outcomes and create efficient treatment protocols for improved patient care of the obese patient population.

## 1. Introduction

Obesity is a growing and significant healthcare issue [1–4]. In 1910, Dr. Kelly described obesity, stating “to be a storehouse for useless adipose tissue and to carry this handicap around, openly displayed wherever one goes, is one of the most distressing of life's minor ailments [5].” While current viewpoints may be less extreme, obesity continues to carry a social stigma and is increasingly present in our society [2, 4, 6, 7]. According to recent figures, an average prevalence of 35 percent of adult Americans is classified as obese and 5 percent morbidly obese, with this trend continuing [2, 8]. Obesity is responsible for more than 25 percent of the increase in healthcare costs over the past 15 years [2, 4].

Body mass index (BMI) is one of the most objective methods in determining the presence and extent of obesity [9–11]. As BMI increases to obesity levels, morbidity and mortality rates rise dramatically [4, 12–20]. The clinical definition for obesity is a BMI ≥ 30 kg/m^2^, with severe obesity defined as a BMI ≥ 35 kg/m^2^ and morbid obesity as a BMI ≥ 40 kg/m^2^ [2]. Patterns of obesity are affected by internal as well as external and environmental factors, including variables such as age, socioeconomic status, ethnicity, and genetic background [2, 3].

Surgical attempts have been made to correct obesity, such as bariatric surgery and direct surgical excision. Bariatric surgery refers to any of the various surgical procedures performed to treat obesity by modification of the gastrointestinal tract to reduce nutrient intake and/or absorption [12, 14, 21–25]. Direct surgical excision, individualized to each patient's needs, may include circumferential abdominoplasty, panniculectomy, brachioplasty, thighplasty, and/or liposuction [7, 26–28]. Usually performed after massive weight loss following bariatric procedures, surgical excision is an important aspect of treatment for many patients as they often view the redundant tissues as the last barrier to resuming “normalcy”, both for cosmetic reasons as well as for physical comfort [29].

From a surgical standpoint, obesity is associated with a multitude of complications including impairments of cutaneous wound healing, total wound failure, and fascial dehiscence [10, 30–39]. Complications can be observed following various surgeries including gastric bypass and panniculectomy [29, 32, 40–42]. While health benefits of bariatric surgery are well recognized, serious complications can result in 2–4% of patients [43]. A 2006 study of postbariatric patients found that over the ensuing six months after surgery, complication rates actually approached 40 percent [2]. These complications included abdominal pain, nutritional deficiencies, endocrine or metabolic disorders, gastrointestinal disorders, and wound compromise. Additionally, patients who have had prior bariatric surgery and subsequently undergo additional surgical procedures may have similar complications as their BMI may persist to be within the obese category [36]. As these postsurgical complications often result in unplanned hospital and emergency room visits, as well as readmissions, there is an imperative need to improve the outcomes of surgery performed on obese patients in order to help decrease health care costs, healing time, and time away from work [2].

Many studies have observed an association between obesity and a myriad of complications [10, 30–39]. Few, however, specifically address the exact mechanisms involved in the deficiencies observed in obese patient wound healing, compared to patients of normal weight. The determination of these mechanisms may allow researchers to pursue a solution, or at least an improvement, to decrease complications and facilitate improved surgical outcomes in the obese patient population.

## 2. Methods

A literature search was performed using PubMed, Medline, the Cochrane Library, and Internet searches. Keywords used included obesity, wound healing, adipose healing, and bariatric and surgical complications. In addition, a manual search for other articles pertinent to the study was performed. A discussion focusing on the effects of obesity on wound healing, specifically in relation to postoperative complications, is presented. Potential etiologic factors involved and current plausible mechanisms are explained.

## 3. Discussion

### 3.1. Complications

Obesity is associated with comorbidities and poor outcomes in a number of medical areas [10, 30–39]. Studies show that even a 10-kilogram weight loss in obese patients can decrease diabetes-related morbidity and mortality by 30% [4, 7, 24]. Improvements are significant in disease processes involving endocrine (diabetes), cardiovascular (hypertension, hyperlipidemia, and coronary artery disease), rheumatic, and hypercoagulation disorders (deep vein thrombosis and pulmonary embolism) [2, 3, 17, 24, 44–46]. Reductions in the severity of symptoms relating to sleep apnea and depression have also been observed [2, 3, 47]. It is important to note that even with the substantial weight loss seen after bariatric procedures, many patients are still considered obese based on their BMI [41, 48].

A higher BMI has repeatedly been correlated with increased mortality resulting from cancers of the uterus, breast, ovary, prostate, colon, and rectum [47, 49]. Baseline values of BMI are an independent predictor of cardiovascular mortality alone [50]. The association of BMI with mortality is further supported by its consistency across different sexes and ethnic groups, with the strength of effect being the main variation [51]. Obese patients suffering a traumatic injury are also at an increased risk of developing multiorgan failure [44]. Since other studies have noted visceral adiposity as having a stronger correlation with increased morbidity and mortality, both BMI and visceral adiposity should be considered as factors that affect the health status of a patient at the time of surgery and during recovery [52–54]. Furthermore, a higher BMI is correlated with increased infection rates in trauma patients [55]. Compared with nonobese surgical patients, obese patients have an increased incidence of surgical complications, including atelectasis, thrombophlebitis, mortality, wound infection, and wound separation [3, 10, 19, 30–32, 34–39, 47, 48, 56–62]. Clearly, there is strong evidence indicating the association between obesity and poorer surgical outcomes, especially in relation to wound healing. However, the mechanisms responsible for these outcomes are not well known nor understood.

Several studies postulate the mechanisms by which obesity increases wound complications. Potential factors include the intrinsic tenuous anatomic properties and poor vascularity of adipose tissue [63–65]. Relative vascular insufficiency, thus decreased oxygen tension, may result in decreased collagen synthesis, decreased capacity to fight infection, and decreased ability to support the necessary mechanisms of the healing cascade [66–73].

### 3.2. Anatomy

Adipose tissue is a distinct entity often perceived as relatively avascular [6, 74, 75]. From this standpoint, ischemic associations are made and used in an attempt to rationalize responses of adipose tissue to various stressors. A study contradicting this theory of avascularity notes the anatomy of adipose tissue is subdivided into lobules, each made up of thousands of cells which are separated by fibroush septa carring relatively large vessels and neurons [64]. The blood supply to each lobule comes through a vascular pedicle, with fine-meshed capillary retia encompassing every fat cell. The percentage of capillary beds to active cytoplasm in fat cells is in fact similar to that of skeletal muscle, a well-vascularized tissue [5, 64]. As excess adiposity causes an increased demand on the circulation, adipose tissue begins to develop its own vascular system. Vascular insufficiencies arise in obesity because the capillary density does not increase proportionally to the increase in adipose tissue [76]. This may be due to several factors, one of which is a decrease in elastin and an increase in collagen V and VI around the large vessels of adipose tissues, resulting in a profibrotic environment [77, 78]. These changes in the extracellular matrix contribute to fibrosis and increased adipose tissue rigidity, likely hindering the expansion of individual adipocytes. This can also potentially restrict capillary proliferation, leading to impaired angiogenesis. Compounding to this fact is the greater number of large blood vessels in obese individuals. Larger vessels have a greater diffusion barrier so they do not deliver oxygen as efficiently as capillaries. Altogether, decreasing capillary density and increasing number of larger vessels lead to decreased perfusion of adipose tissue and predispose it to a hypoxic environment. This could be a significant detractor for vascular regeneration and oxygen perfusion needed in wound healing. While the strain on circulation created by surplus adipose mass already exists, capillary damage from injury further exacerbates this hypoxic environment.

Structurally, the delicate nature of fat lobules makes it more susceptible to mechanical damage—the *in-situ* resistance ascribed to only the supporting structures [64]. These supportive structures, however, do not develop correspondingly to increasing fat volume [64]. Surgical intervention can render these supportive structures inactive and readily cause adipose weakness. Additionally, fat lobules are units with terminal circulation and severance of the lobule may destroy blood supply, leading to necrosis of the whole unit [64, 65, 73]. Capillary proliferation occurs maximally at the wound surface during normal wound healing. However, necrosed or damaged adipose lobules can complicate this process [31, 64, 79]. With full-thickness skin grafts, complications arise when residual adipose tissue is attached to the graft [64, 65, 80]. Adipose tissue appears to inhibit the flow of wound serum into grafted skin, a process essential to the graft's survival. It is postulated that this interaction is due to the adipocytes' absorption of wound serum with failure to subsequently transfer it to the graft, a theory supported by the substantial edema noted in grafts containing residual adipose tissue, and decreased revascularization of these grafts since fewer capillary links with the dermis exist among the vascular anastomoses between the wound base and the lobules [64, 80]. Decreased angiogenic capacity of adipose tissue in conjunction with decreased oxygenation of graft tissue can lead to hypoxia.

Adipose tissue grafting is notable for variable and often unsatisfactory results due to loss of volume. Fat lobules damaged or severed on removal of the graft are likely to be lost [64]. Rapid revascularization would involve using original vessels, but due to the anatomical features of adipose tissue, this can only occur indirectly. Indirect revascularization possibly results in substantial delay and thus, increased loss of cells incapable of tolerating the ischemic stress as hypoxia may be a risk factor for increased adipocyte death in obese patients [64, 65, 73, 81]. Once becoming necrotic, fat grafts cannot be resorbed but rather need macrophage uptake for elimination [64, 65]. Macrophage recognition of necrotic adipose as a “foreign body” may lead to initiation or perpetuation and extension of the inflammatory phase of an acute or chronic wound. This reaction can be detrimental to ideal healing processes as “foreign bodies” delay the progression of wounds to the fibroproliferative phase, a critical wound healing period where tensile breaking strength quickly increases [61, 82]. Fat necrosis and subsequent foreign body reaction can be substantial if surgical manipulation is not performed along anatomical limits of the fat layer [64].

### 3.3. Vascularity

Several studies describe the “inherent” decreased vascularity of adipose tissue as leading to its associated wound complications [6, 74, 75]. Increased adiposity sets off a cascade that positively feeds back into impaired angiogenesis and chronic low-grade inflammation. In addition to the overexpression of collagen VI (col6), obesity is associated with increased expression of 11f3-hydroxysteroid dehydrogenase type 1 (11f3HSD1), an intracellular glucocorticoid-amplifying enzyme [83]. Glucocorticoids suppress angiogenesis so the elevated level of 11f3HSD1 amplifies the inhibitory effects of glucocorticoids [84, 85]. As adipose tissue becomes hypoxic from impaired angiogenesis, hypoxia-inducible factor 1 alpha (HIF1**α**) levels increase [86]. Elevated HIF1**α** levels initiate local inflammation and fibrosis by activating the collagen I and III cross-linking activity of lysyl oxidase (LOX). Consequently, overexpression of these factors impairs wound healing by suppressing the angiogenic process needed to restore the wound vasculature and contributes to the obesity pathogenesis of hypoxia and fibrosis. Other proposed mechanisms in which excessive adiposity leads to microvascular abnormalities include reduced nitrogen oxide (NO) availability impairing development of microvascular rarefaction and prolonged elevation of free fatty acids in the blood from increased fat mass impairing capillary recruitment [76, 87, 88].

Yet, a few studies debate this viewpoint of inherent decreased vascularity, pointing to the finding that undisturbed adipose is well perfused [58, 64, 65]. Only in particular scenarios is decreased vascularity observed, such as surgical and other traumatic injuries that result in adipose lobule disruption and consequent relative avascularity [64]. In response to ischemic stimulation, obesity in murine models suppresses the ability of adipose-derived endothelial progenitor cells (EPC) to differentiate, reducing the circulating level of EPCs available to function in blood vessel endothelial repair and angiogenesis [89]. Regardless of whether the avascularity is inherent or acquired, the compromise of blood flow and subsequent poor oxygenation can lead to disruption of the normal healing process and/or eventual necrosis of the tissue [64, 90, 91].

Venous insufficiency is another vascular factor associated with the development of chronic wounds and delayed wound healing. The association between obesity and venous insufficiency is well established, but the exact mechanisms for wound disturbances are only beginning to be realized. Understanding of the more advanced clinical stages of venous disease in obese patients has led to a proposal in which raised intra-abdominal pressure may cause the greater reflux, increased vein diameter, increased venous pressure, and ultimately, impaired venous function [92]. Venous insufficiency impairs wound healing by forming a barrier surrounding capillaries and decreasing effective diffusion of oxygen and nutrients from capillaries to surrounding tissue [66–68, 71, 73]. Once venous insufficiency is present, proteinaceous material may accumulate in the interstitium surrounding the capillaries, leading to clotting and eventual fibrosis of these vessels [93, 94]. Additionally, components of intravascular fluid may leak into the surrounding tissues because of increased hydrostatic pressure, inciting an inflammatory response [95]. These factors create a “barrier” through which oxygen and nutrients cannot transverse to supply surrounding tissue. Furthermore, leukocytes may become trapped and accumulate, contributing to poor oxygenation and tissue destruction by releasing lysosomal enzymes and proinflammatory mediators [93, 94]. These mechanisms create a feasible argument for the role of venous insufficiency in contributing to impaired wound healing.

### 3.4. Cellular and Molecular Alterations

Advances in the field of gene array offer a potential to investigate the changes corresponding with obesity [96]. The discoveries of obesity-induced changes in adipose tissue emphasize that obesity may initiate and perpetuate a chronic low-grade inflammatory process. A study of obese mice demonstrated a progressive increase in proinflammatory cytokine production resulting from the activation of invariant natural killer T (iNKT) cells by the excess lipid. This occurred before or at the time of inflammatory leukocyte recruitment [97]. Furthermore, the iNKT cells influenced other leukocytes to produce proinflammatory cytokines, resulting in an imbalanced proinflammatory cytokine milieu. Other inflammatory mediators that increase concomitantly with adipose tissue mass include angiotensinogen, tumor necrosis factor alpha (TNF-**α**), leptin, interleukin 6 (IL-6), and transforming growth factor beta (TGF-**β**). [1] Additionally, pathologic inflammatory changes related to increased macrophages, mononuclear and polymorphonuclear cells, and inflammatory cytokines have been shown in obesity studies to be dependent upon the degree and duration of obesity [1, 96, 98, 99]. [1] As lipopolysaccharide levels increase, the scavenger receptor CD36 enhances adipose tissue inflammation and cell death through its expression on and synergistic modulation of both macrophages and adipocytes [100]. Specifically, the activation state of adipose tissue macrophages shifts from the protective and anti-inflammatory M2-polarized state to an M1 proinflammatory state as a result of diet-induced obesity [101]. M2 macrophages aid in tissue repair and homeostasis and produce anti-inflammatory cytokines [102]. In obese mice, chronic inflammation caused by mediators such as TNF-**α** can cause skin gamma delta T cells to be unresponsive to damage in the epithelium, preventing the release of cytokines and growth factors that facilitate wound repair and making the wound vulnerable to injury and infection [103]. Thus, the shift in macrophage activation and other immune mediators towards a proinflammatory state likely impairs wound healing in obese patients by compromising immunoregulation and altering the inflammatory stage of wound repair. The view that obesity induces a chronic low-grade inflammatory process is supported by findings of a reduction in macrophage infiltration and chemoattractant gene expression following weight loss [1, 96].

### 3.5. Oxidative Stress

Obesity, especially abdominal obesity, is positively correlated with oxidative stress [104]. This is partly attributed to a deficiency in adiponectin, an adipose-derived cytokine that protects against oxidative stress and inflammation [105, 106]. Paradoxically, adiponectin concentration decreases with obesity although it is secreted by adipocytes [107]. A deficiency of adiponectin impairs wound healing via two mechanisms. In response to ischemic stimulation, adiponectin stimulates angiogenesis by promoting AMP-activated protein kinase signaling. Secondly, adiponectin activates the ERK signaling pathway to promote keratinocyte proliferation and migration, a critical process in the reepithelialization phase of wound healing [108]. Therefore, decreased adiponectin impairs proper perfusion and reepithelialization of the wound.

Decreased oxygen tension is present in “normal” wounded tissue and is a major factor in the susceptibility of wounds to infection [66, 67, 69, 109, 110]. Baseline oxygen tension was noted to be the lowest in infected wounds, with the most critical range being between 0 and 40 mmHg [71, 73]. The hypovolemia and impaired tissue perfusion of the wound may exacerbate the preexisting relative hypoperfusion of adipose tissue that resulted from the expansion of fat mass without a synchronized increase in blood flow per cell [111]. This can cause a critical reduction in oxygen delivery to adipose tissue, as seen in the impaired tissue perfusion of the morbidly obese during the perioperative period [88].

The hypoperfusion of subcutaneous adipose tissue in obese patients may predispose them to a greater risk of acquiring a surgical site infection [110]. This is likely from a greater risk of ischemia and necrosis and deficiencies in the oxidase system of leukocytes [69, 112, 113]. In the normal, unhindered process, phagocytosis is followed by a burst of oxygen consumption, which coincides with bacterial death [68, 69, 71, 112]. This oxygen consumption is essential to the bactericidal mechanisms of the host's defense system. In an oxygen deficient setting, leukocytes can ingest the bacteria but are unable to kill them [71]. Bacteria cultured from infected wounds of obese patients have been noted to be similar to longstanding wounds of chronic granulomatous disease patients, where the main issue is a defective oxidase system (*i.e., Staphylococcus, E. coli, Klebsiella,* and *Candida)* [68, 70, 71, 112, 114]. The positive feedback interaction between the altered immune responses and local inflammation in adipose tissue contributes to the development of metabolic complications and impaired wound healing in the obese [115].

Infection significantly affects wound healing and preparation of the wound bed. Optimal healing requires eradication of infection [82, 116–119]. Therapies with proven efficacy incorporate minimizing necrosis, bacterial load, and inflammation of the wound bed, all the while maintaining regenerative tissue cells to aid in wound healing [120]. A fine balance exists between the host's defense system and the pathologic effects of bacteria [82, 119, 121]. The faster and more effectively the immune system combats infection, the less disturbed the normal wound healing process and the better the outcome for the patient. Deficiencies in oxygen utilization and increased inflammation associated with adipose tissue partially explain the mechanisms of increased wound infections prominent in obese patients.

Another aspect of wound healing that is potentially affected by hypoxia is fibroblasts' capability to synthesize collagen [73, 93, 94]. Although fibroblasts are able to survive in a hypoxic environment, they cannot proliferate or synthesize collagen properly [73]. Biochemically, the complete absence of oxygen inhibits the hydroxylation of proline and lysine, preventing the final assembly of the collagen molecule [122]. Clinical support of this is evident in the finding of collagen deposition and synthesis being directly proportional to oxygen tension in postsurgical patients [67, 68]. As a result, injured tissues, especially damaged adipose lobules that already have an insufficient vascular supply and experience further oxygen perfusion deficits after an injury, have decreased resources to meet metabolic needs and have a limited ability to effectively produce collagen. Without sufficient collagen IV and V to promote the aggregation of platelets in the subendothelium, hemostasis is delayed and/or impaired [123]. This likely translates into poor wound healing.

Collagen synthesis is an essential part of the wound healing process and subsequent integrity of the wound [124–127]. It is a key element in establishing maximum tensile strength [67, 124–127]. The upregulation of collagen I and III expression has been shown to accelerate cutaneous wound healing in rats [128]. This process is impaired in obesity because excessive adiposity increases the expression of immature type III collagen and disorganized type I collagen fibers [129]. Without the appropriate collagen, the collagen matrix cannot remodel properly, which reduces the mechanical strength and yield required for the recovery of wound strength and healing.

### 3.6. Micronutrient and Macronutrient Deficiencies

Wound healing involves the well-coordinated processes of hemostasis, inflammation, granulation tissue formation, fibrogenesis, neovascularization, contraction, and reepithelialization [130]. In order for the cellular responses and homeostatic mechanisms to occur properly, there must be sufficient vitamins, minerals, and proteins present, that is, there must be sufficient nutrients available. This requirement necessarily places obese patients at a disadvantage because they suffer from a paradoxical malnutrition resulting from a calorie-dense diet that is high in carbohydrates and fats and low in vitamins and minerals [131]. Eventually, nutritional deficiencies occur. Blood tests and biochemical analysis on bariatric surgery candidates preoperatively revealed an astonishingly high occurrence of micronutrient and macronutrient deficiencies, particularly vitamin B12, 25-OH vitamin D3, zinc, albumin, and iron, with the latter two increasing significantly with BMI [132–136]. Other nutritional deficiencies amongst bariatric surgery candidates include folic acid, ferritin, phosphorus, calcium, magnesium, vitamin A, vitamin B6, vitamin C, and copper [133–135, 137, 138].

Almost immediately after the occurrence of a wound, hemostasis begins to take place. Calcium, predominantly involved as factor IV, is critical in this process as it is required for the activation of many intercellular reactions, serving as a primary catalyst in platelet aggregation and in the production of clotting Factors VIII, IX, and X [123, 139]. Calcium is recruited again later on for the activation of neutrophils, migration of cells, regeneration of epidermal cells, and modulation of keratinocyte proliferation and differentiation. Without sufficient vitamin D, however, calcium cannot be absorbed in the small intestines [140]. Vitamin D deficiency is also associated with obesity [141, 142]. BMI may be a risk factor for vitamin D deficiency, with deficiencies even evident in obese children [143]. Vitamin D has a range of physiological functions, from regulating B cells and bone metabolism to maintaining calcium homeostasis [141, 144]. The role of vitamin D in adipose tissue signaling is being unraveled, with recent findings of vitamin D reducing lipid accumulation in adipocytes and exhibiting an anti-inflammatory effect in adipose tissue [144]. This implies that vitamin D has an immunomodulatory function in adipose tissue and deficiencies may lead to adipocyte dysfunction and impair wound healing.

Proper wound healing also requires the minerals zinc and copper. DNA and RNA polymerases require zinc in the repair processes as it helps the metabolism catalyze more than 200 enzymes, facilitates protein folding, and regulates gene expression [123, 145]. Adipose tissue metabolism requires zinc to regulate the secretion of leptin, an adipocytokine, and promote free fatty acid release and glucose uptake [146]. Obesity is associated with leptin resistance; thus, the abnormal metabolism resulting from zinc deficiency may contribute to impaired wound repair [106]. During the inflammatory and fibrogenesis processes, zinc and zinc metalloenzymes assist in immune and inflammatory responses, collagenesis, matrix degradation, epidermal regeneration, and scar formation [123, 146, 147]. Low zinc intake is associated with low superoxide dismutase activity in obese individuals, likely accounting for why zinc deficiency increases oxidative stress and the inflammatory response [148]. In a clinical study, oral administration of nutritional doses (15 mg elemental zinc) of zinc to burn patients significantly improved outcomes by elevating the level of glutathione (a natural antioxidant), decreasing the incidence of eschar formation, improving the antioxidant status, increasing healing time, and decreasing the mortality rate [149]. In addition, copper is needed by lysyl oxidase in collagenesis and elastic tissue formation and may also have a role in stabilizing epidermal cells [123].

The inflammatory stage may be prolonged if vitamin A is deficient as low vitamin A serum levels correlate with increased oxidative stress and Th1 response, which potentially can heighten the inflammatory processes already involved in the chronic inflammation and fat deposition inherent in obesity [148]. Vitamin A is needed again in collagenesis and epithelialization to increase the rate of collagen synthesis, cross link new collagen, promote epithelialization, and close wounds [23, 150, 151]. Experimental animal studies have shown that vitamin A accelerates the healing of incisions by hastening the formation of granulation tissue [150, 152]. Vitamin A also has the capability of decreasing the severity of bacterial and fungal infections [151]. Due to the increased nutritional requirements for wound healing, it is recommended to give five times the normal supplemental vitamin. A requirement to all patients after an injury, regardless of the patient's nutritional status [150, 151]. Vitamin A can also reverse the negative effects of steroids on wound healing so it may contribute to improved wound healing in various compromised states, for instance, obesity [153–155].

Throughout the process of wound healing, there are also other nutritional factors that are required, such as vitamin B. B complex vitamin deficiencies are concerning as they influence wound healing and other processes [94, 156–160]. B12 injections have been shown to increase the tensile strength of wounds and facilitate superior wound healing [82, 158]. Components of vitamin B complex also serve as cofactors for a number of enzyme systems. Dysfunction of these enzyme systems can affect protein, carbohydrate, and fat metabolism, as well as disturb antibody and white cell functions [70, 157, 158, 160]. Severe deficiencies impair the synthesis of DNA, proteins, and other crucial molecules, leading to megaloblastosis as well as hindering cellular proliferation and repair [161]. Furthermore, megaloblastosis can result in the development of megaloblastic anemia, which may adversely affect circulation. To maintain sufficient vitamin B intake for patients who are severely injured or acutely ill, five to ten times the normal amount of B complex vitamins is usually recommended [64].

Iron is also crucial for proper wound healing. Iron deficiency in the obese may be due to a decrease in dietary iron intake, the effects of obesity-related inflammation on dietary iron absorption, and/or the sequestration of iron as a result of chronic inflammation [148, 162]. Both BMI and inflammation have been shown to predict iron absorption and affect iron fortification response [163]. It is plausible that hepcidin, an inhibitor of intestinal iron absorption and activator of iron sequestration by macrophages, is a mediator as it is directly correlated with the degree of obesity and indirectly correlated with iron absorption [148, 164, 165]. Iron is also required in the hydroxylation of lysine and proline; therefore, insufficiencies of this element can lead to decreased tensile strength in wounds [166, 167]. Additionally, decreased iron absorption may result in iron deficiency anemia. This can diminish oxygen transport and potentiate secondary effects on wound healing due to relative hypoxia in healing tissues [150, 167, 168]. Iron deficiency also results in a dampened leukocyte bactericidal ability, further complicating healing by weakening the wound's defense system [169].

Protein malnutrition also significantly affects wound healing because it is needed for fibroblast proliferation, angiogenesis, and collagen production [150, 161]. An albumin level lower than 2 is associated with a number of wound healing impairments including a prolonged inflammatory phase, decreased fibroplasia, which leads to delayed wound healing, and decreased neovascularization [150]. Additionally, decreased cell synthesis and wound remodeling, diminished wound strength, impaired T-cell function, and phagocyte activity, as well as decreased complement and antibody levels, all contribute to the delayed healing observed in protein deficient states [170–173].

Carbohydrates and fat intake also affect wound metabolism [150, 174]. Together, carbohydrates and fats are the major source of energy in the body and consequently in the wound healing process. The metabolic syndrome commonly associated with obesity may lead to insulin resistance, rendering the body less adept at maximally utilizing glucose, a breakdown product of carbohydrates [175]. Indirectly, insufficient carbohydrates and/or fats will force the body to look for alternate forms of energy, leading to excess oxidation of amino acids to satisfy caloric needs [150]. Following injury, obese patients preferentially oxidize proteins and carbohydrates, possibly from a block in the utilization of free fatty acids [176]. Amino acid use further perpetuates protein malnutrition and their effects on wound healing. Directly, the energy requirement of leukocytes and fibroblasts is increased after injury, and if unfulfilled, can result in impaired wound healing [150]. This altered metabolic function after trauma and injury can reduce the immune response. Cumulatively, all these factors contribute to increasing wound complications and wound failure rates.

### 3.7. Special Considerations

Gastric bypass (GBP) surgery can be utilized to correct obesity by helping patients achieve and maintain weight loss [2, 36, 47]. An important topic often left unaddressed with patients, however, is the increased risk of possible wound complications following surgery. The nature of GBP surgery leaves patients in a state of “deliberate malnutrition,” possibly exacerbating the altered metabolic response and nutritional deficiencies already present in the obese patient. GBP patients, in addition to the normal challenge of maintaining the 25% increase in required protein and caloric intake for wound healing postoperatively, are faced with a new and less effective gastrointestinal anatomy for maintaining the nutrients needed postoperatively [150]. Many post-GBP patients have protein-calorie malnutrition in addition to various vitamin and mineral deficiencies. [177] Post-GBP nutritional deficits in iron, vitamin B12, folate, vitamin A, thiamine, calcium, potassium, and magnesium are relatively common [178, 179]. Following major elective surgery, additional nutritional deficits include reductions in nitrogen, potassium, sulfur, and phosphorus [150]. These deficits are compounded by postsurgical requirements for increased ascorbic acid, thiamin, riboflavin, nicotinamide, and vitamin A, compared to normal daily needs. This further reduction in the quantity of nutrients available for use can lead to an increased risk of medical complications [20, 174]. In a study of postbariatric patients, all of them experienced wound complications following abdominoplasty because they had a reduction of nearly 60% for total tissue protein [177]. These same patients had a reduction of 72% in the amino acid hydroxyproline, a major constituent of collagen, leading to a decrease in tensile strength of the wound. However, a reduction in surgical complications is achievable by giving nutritional supplements to obese patients prior to surgery. Dietary recommendations suggest that unsaturated fatty acids comprise 4–8% of daily caloric intake in injured patients who experience metabolic/physiologic derangements that are prolonged (as in post-GBP patients) [150]. This amount is roughly double the amount recommended for healthy patients [94, 158, 159]. Diet adjustments of post-GBP patients that subsequently undergo panniculectomy or other surgical procedures must be quantified and supplemented in order to maintain sufficient nutritional status. The inability to provide for these requirements can significantly affect patient outcome. Vitamin and protein deficits diminish phagocytic and lymphocytic activity, directly impairing the immune system, after only 4 weeks in this nutritional setting [82, 116, 118]. While nutrition teams closely follow patients postoperatively and advances in supplementation attempt to correct nutritional deficiencies after surgery, the system is by no means perfect as patient noncompliance can negatively impact nutritional and metabolic complications [2, 20, 47, 174].

## 4. Conclusion

Obesity is a pervasive health issue in today's society. It induces complex negative effects on multiple organ system functions and processes, including issues related to wound healing. Efforts to decipher the origins, physiology, and health consequences of obesity on the human body are an imperative focus in current investigations. Numerous studies have shown the correlation between obesity and abnormal wound healing, with knowledge of the exact mechanisms in the infantile stages. Theories on credible contributing factors are presented in this review.

Healthy adipose tissue is beneficial to the human body as it serves as an insulator and paracrine and endocrine organ, secreting cytokines, growth factors, and immune mediators. Uninjured adipose tissue has a comparable vascular supply to other uninjured tissue types, but its tenuous anatomic configuration leaves it weaker and more susceptible to mechanical damage. Since each lobule is supplied by terminal blood vessels, damage to the area may result in necrosis of the entire lobule.

Problems arise as excessive adiposity modifies the cellular composition and structure of adipose tissue, as well as changing the physiology of the skin and subcutaneous tissues. Obesity induces adipocyte hypertrophy and hyperplasia, eventually impairing the metabolic functions of the adipocytes, such as storing fat. It may be that the metabolic dysfunction is the primary activator in the cascade of obesity-induced problems. Following metabolic dysfunction, inflammatory mediators begin to invade the adipose tissue, leading to a chronic, low-grade inflammatory process that is well associated with obesity. The phenotypic switching of protective M2 macrophages to proinflammatory M1 macrophages exacerbates this problem. As adipocytes grow in size and number, blood vessels develop into the new areas of adipose tissue growth. However, the rate of angiogenesis does not parallel the rate of adipocyte growth. Compounding these properties, obese adipose tissues secrete angiogenic inhibitors and fibrotic mediators. The extracellular matrix remodeling may further impede the process of angiogenesis by creating a stiffer environment and preventing the migration of cells and vessels. Without sufficient supply of blood vessels to oxygenate the area, relative hypoxia ensues. The hypoxia caused by damage to capillaries in wounds and the relative hypoxia in obese individuals likely contribute to higher rates of wound infections in obese patients because of even lower oxygen tension from decreased perfusion and impaired immune system functioning. Additionally, hypoxic wounds impair the synthesis of mature collagen, leading to weaker tissue and deficiencies in the overall healing process. Thus, the microvascular abnormalities as a result of excessive adiposity contribute to obesity-related microangiopathy.

The vascular insufficiencies and altered population of immune mediators present may lengthen the inflammatory stage of wound healing, as well as leaving obese individuals more susceptible to infections. Wound healing is also delayed as a result of macronutrient and micronutrient deficiencies in obese individuals. Without the proper cofactors and enzymes, the process of wound healing is compromised, as well as the integrity of the wound. Nutritional supplements can be given to obese patients preoperatively as a possible solution to decrease wound complications. For bariatric surgery candidates, special considerations need to be made as they are at increased risk of nutrition-related complications.

Although the detrimental effect of obesity on wound healing is established, there is a need for additional studies to determine the comprehensive mechanisms involved in this relationship. Some of the key alterations and deficiencies in obese adipose tissue that already have been determined are presently undergoing investigations that utilize target-specific therapies. Perhaps the breakthrough with obesity and wound healing will be in the realm of growth factors, cytokines, nutritional manipulations, surgical techniques, or in gene array studies. Most likely, interventions will involve a combination of these factors and others. With the prevalence of obesity in today's society and its evidenced effects on health care, the challenge of altering adipose tissue's effect on wound healing remains one which necessitates further exploration.
